# Detection of *Toxoplasma gondii* DNA in horse meat from supermarkets in France and performance evaluation of two serological tests

**DOI:** 10.1051/parasite/2015014

**Published:** 2015-03-25

**Authors:** Abdelkrim Aroussi, Philippe Vignoles, François Dalmay, Laurence Wimel, Marie-Laure Dardé, Aurélien Mercier, Daniel Ajzenberg

**Affiliations:** 1 INSERM, Univ. Limoges, CHU Limoges, UMR-S 1094, Laboratoire de Parasitologie 87000 Limoges France; 2 INSERM, Univ. Limoges, CHU Limoges, UMR-S 1094, Institut d’Epidémiologie Neurologique et de Neurologie Tropicale 87000 Limoges France; 3 Institut Français du Cheval et de l’Équitation, Station Expérimentale, Domaine de la Valade 19370 Chamberet France; 4 Toxoplasma Biological Resource Center, CHU Limoges 87042 Limoges France

**Keywords:** *Toxoplasma gondii*, Horse meat, Enzyme-linked immunosorbent assay, Modified agglutination test, Magnetic-capture polymerase chain reaction

## Abstract

In France, some cases of severe toxoplasmosis have been linked to the consumption of horse meat that had been imported from the American continent where atypical strains of *Toxoplasma gondii* are more common than in Europe. Many seroprevalence studies are presented in the literature but risk assessment of *T. gondii* infection after horse meat consumption is not possible in the absence of validated serological tests and the unknown correlation between detection of antibodies against *T. gondii* and presence of tissue cysts. We performed magnetic-capture polymerase chain reaction (MC-PCR) to detect *T. gondii* DNA in 231 horse meat samples purchased in supermarkets in France and evaluated the performance and level of agreement of the modified agglutination test (MAT) and enzyme-linked immunosorbent assay (ELISA) in the meat juices. The serological tests lacked sensitivity, specificity, and agreement between them, and there was no correlation with the presence of *T. gondii* DNA in horse meat, raising concerns about the reliability of *T. gondii* seroprevalence data in horses from the literature. *T. gondii* DNA was detected in 43% of horse meat samples but the absence of strain isolation in mice following inoculation of more than 100 horse meat samples suggests a low distribution of cysts in skeletal muscles and a low risk of *T. gondii* infection associated with horse meat consumption. However, to avoid any risk of toxoplasmosis, thorough cooking of horse meat is recommended.

## Abstract (fr)

En France, quelques cas de toxoplasmose sévère ont été liés à la consommation de viande de cheval qui avait été importée du continent américain où les souches atypiques de *Toxoplasma gondii* sont plus fréquentes qu’en Europe. De nombreuses études de séroprévalence existent dans la littérature mais l’estimation du risque d’infection par *T. gondii* après consommation de viande de cheval est impossible à cause de l’absence de validation des tests sérologiques et la corrélation inconnue entre la détection des anticorps anti *T. gondii* et la présence de kystes dans les tissus. Nous avons utilisé la technique de capture magnétique-réaction de polymérisation en chaine (MC-PCR) pour détecter l’ADN de *T. gondii* dans 231 échantillons de viande de cheval achetés dans des supermarchés en France et évalué la performance et le niveau d’agrément du test d’agglutination modifié (MAT) et de la méthode immuno-enzymatique ELISA dans les jus de viande. Les tests sérologiques manquaient de sensibilité, de spécificité, d’agrément entre eux et il n’y avait pas de corrélation avec la présence d’ADN de *T. gondii* dans la viande de cheval, ce qui suscite des doutes sur la fiabilité des données de séroprévalence de *T. gondii* chez les chevaux dans la littérature. L’ADN de *T. gondii* a été détecté dans 43 % des échantillons de viande de cheval mais l’absence d’isolement de souche chez la souris suggère une faible répartition des kystes dans les muscles squelettiques et un faible risque d’infection par *T. gondii* avec la consommation de viande de cheval. Cependant, pour éviter tout risque de toxoplasmose, il est recommandé de bien cuire la viande.

## 1. Introduction

The intracellular protozoan *T. gondii* is able to infect all warm-blooded animals worldwide, including humans. It is estimated that one third of the world’s population is chronically infected by this highly successful parasite. Humans can be infected by tachyzoites vertically during pregnancy but are more often infected later in life by bradyzoites after ingestion of tissue cysts in raw or undercooked meat, or by sporozoites after consumption of vegetables or water contaminated by oocysts in cat feces. Between 30 and 63% of *T. gondii* infections are attributed to consumption of tissue cysts in infected raw or undercooked meat [8]. Tissue cysts are the terminal life-cycle stage in the intermediate host; they are immediately infectious, and are able to remain quiescent for a very long period of time [39].

In France, after episodes of trichinellosis outbreaks and *Salmonella* poisoning due to horse meat consumption, the government implemented measures to protect consumers, such as prohibiting horse meat in schools (Interministerial circular of March 6, 1968). *T. gondii* has been isolated from meat cuts of many naturally infected animal species but tissue cysts are less frequently present in infected horses [2]. In France, some cases of severe toxoplasmosis infection due to consumption of imported horse meat have been described and genetic analyses of *T. gondii* strains revealed atypical genotypes that are highly uncommon in Europe [19, 34, 37]. Accurate evaluation of the risk of *T. gondii* infection after horse meat consumption should rely on reliable serological data for *T. gondii* infection in horses. Since the major review by Tassi in 2007 [38], additional seroprevalence data for *T. gondii* in horses has become available in the literature [3–6, 9, 20, 22, 24–28, 30, 31, 40]. One remarkable finding about *T. gondii* seroprevalence in horses in this abundant literature is its high variability between countries, from 1 to 90%. This prevalence may also vary widely within the same country: 13–67% in Argentina, 14–53% in Belgium, 5–90% in Brazil, 17–80% in Italy, and 4–55% in Switzerland [30]. This variability in seroprevalence may have biological and epidemiological explanations, such as differences in the age of horses, their environmental lifestyle, the abundance of oocysts in the soil, and hygiene standards on farms [39]. However, an alternative explanation for the variability of *T. gondii* seroprevalence in horses may simply come from the absence of a gold standard for detecting *T. gondii* antibodies in horses and the lack of validation of the serological methods used in all these studies. The absence of a gold standard is evidenced by the diversity of serological tests used in the literature, such as enzyme-linked immunosorbent assay (ELISA), direct agglutination test (DAT), indirect immunofluorescence antibody test (IFAT), indirect hemagglutination test (IHAT), latex agglutination test (LAT), modified agglutination test (MAT), and Sabin-Feldman dye test (SFDT). The lack of validation is shown in the literature by the use of different cut-offs for a single test, without any data on sensitivity, specificity, and agreement between tests.

Our main objective was to detect *T. gondii* DNA by magnetic-capture polymerase chain reaction (MC-PCR) in horse meat destined for human consumption and to evaluate the performance of MAT and ELISA serological tests in meat juices of these samples.

## 2. Materials and methods

### 2.1. Samples

A total of 231 horse meat steak samples were purchased from supermarkets in several cities in France from November 2012 to April 2014. At least 150 g per meat sample was required to perform all tests (50 g for mouse bioassay and 100 g for MC-PCR). Data including lot number, geographic origin, packaging, and expiration date were collected for all horse meat samples, but the date of horse slaughter and geographic origin were not always available in the retail meat packaged for sale in the supermarkets. Horse meat samples were analyzed as quickly as possible to maximize the viability of *T. gondii* that might be present in muscles. Meat juices were directly recovered or obtained after a freezing/thawing step, allowing the muscles to release juice, then stored at −20 °C until tested by MAT and ELISA.

A positive serum sample (THI1813) from a pony experimentally infected with *T. gondii* oocysts in 1985 was kindly provided by Dr. Dubey [11]. The MAT titer of this sample was positive up to dilution 1:102,400 [15].

### 2.2. Magnetic-capture polymerase chain reaction (MC-PCR) of *T. gondii* DNA

The MC-PCR test involved preparation of crude DNA extract from 100 g samples of horse meat, magnetic capture of *T. gondii* DNA, and quantitative real-time PCR targeting the *T. gondii* 529 bp repeat element as previously described [32].

### 2.3. Mouse bioassay

A mouse bioassay was also attempted for horse meat samples with a positive MC-PCR test result and in some samples negative with this test. Two horse meat samples with a positive MC-PCR result were not inoculated because of an excessive delay between MC-PCR and bioassay. Swiss mice weighing 20–25 g were used in the bioassay. Fifty grams of horse meat samples was digested as previously described [14] and 0.5 mL of each digested homogenate was intraperitoneally inoculated into three mice per sample. Mice blood samples were collected 3–4 weeks later and the sera were tested for specific *T. gondii* antibodies by MAT. All procedures in mice were carried out in compliance with ethical rules and approved by the Regional Ethics Committee Limousin (Registration Number: CREEAL 3-07-2012).

### 2.4. Modified agglutination test (MAT)

Both *T. gondii* RH strain antigen preparation and MAT test were performed as previously described [10]. The test was considered positive when a layer of agglutinated parasites formed in wells. Meat juices were tested at five serial dilutions (1:10, 1:20, 1:40, 1:100, and 1:400). The cut-offs for MAT were designated as MAT > 0, MAT > 1:10, MAT > 1:20, and MAT > 1:40. At the MAT > 0 cut-off, the positive samples were those with a test positive at dilutions 1:10, 1:20, 1:40, 1:100, and 1:400. At the MAT > 1:10 cut-off, the positive samples were those with a test positive at dilutions 1:20, 1:40, 1:100, and 1:400. At the MAT > 1:20 cut-off, the positive samples were those with a test positive at dilutions 1:40, 1:100, and 1:400. And lastly, at the MAT > 1:40 cut-off, the positive samples were those with a test positive at dilutions 1:100 and 1:400.

### 2.5. Enzyme-linked immunosorbent assay (ELISA)

Horse IgG antibodies to *T. gondii* were tested by using the commercial kit multi-species ID Screen^®^ Toxoplasmosis Indirect (IDVet, Montpellier, France). The test procedure was performed according to the manual provided by the manufacturer. The optical density (OD) values were read at 450 nm using a microplate spectrophotometer Thermo Scientific™ Multiskan™ GO.

### 2.6. Statistical analysis

Cut-off values for MAT and ELISA were selected by receiver operating characteristic (ROC) analysis. In horse meat juices, the cut-offs were estimated by using MC-PCR as the reference test. For the commercial ELISA test, the cut-off was also determined by testing several dilutions of the positive horse serum THI1813. Comparisons of matched pair results between two tests with the selected cut-offs were performed by the McNemar test. Kappa coefficient was used to estimate agreement between tests for the detection of *T. gondii* infection in meat juice.

## 3. Results

### 3.1. Sampling data and test results

Most of the horse meat sampled in this study was from the Americas, with three major export countries: 40% from Argentina, 20% from Canada, and 14% from Mexico (Table 1). Full data including the list of horse meat samples with geographic origin and test results are available in the Supplementary material.

**Table 1.** Geographic origin of 231 horse meat samples and prevalence estimation of *T. gondii* in horse meat according to MC-PCR, MAT, and ELISA (OD) results.

Country No. (%)	MC-PCR + No. (%) [95% CI]	MAT > 0 No. (%) [95% CI]	MAT > 1:20 No. (%) [95% CI]	MAT > 1:40 No. (%) [95% CI]	OD > 0.06 No. (%) [95% CI]	OD > 0.1015 No. (%) [95% CI]	OD > 0.1145 No. (%) [95% CI]	OD > 0.15 No. (%) [95% CI]
Germany	1 (50.00%)	0 (0.00%)	0 (0.00%)	0 (0.00%)	2 (100.00%)	2 (100.00%)	2 (100.00%)	2 (100.00%)
2 (0.86%)	[0.01–0.98]	[0–0.842]	[0–0.84]	[0–0.84]	[0.15–1]	[0.15–1]	[0.15–1]	[0.15–1]
Argentina	50 (53.76%)	56 (60.22%)	18 (19.35%)	12 (12.90%)	81 (87.10%)	52 (55.91%)	52 (55.91%)	40 (43.01%))
93 (40.26%)	[0.43–0.64]	[0.49–0.70]	[0.11–0.28]	[0.06–0.21]	[0.78–0.93]	[0.45–0.66]	[0.45–0.66]	[0.32–0.53]
Canada	12 (26.09%)	31 (67.39%)	17 (36.96%)	8 (17.39%)	44 (95.65%)	30 (65.22%)	29 (63.04%)	25 (54.35%)
46 (19.92%)	[0.14–0.41]	[0.52–0.80]	[0.23–0.52]	[0.07–0.31]	[0.85–0.99]	[0.49–0.78]	[0.47–0.76]	[0.39–0.69]
France	10 (71.43%)	10 (71.43%)	1 (7.14%)	0 (0.00%)	12 (85.71%)	7 (50.00%)	6 (42.86%)	4 (28.57%)
14 (6.06%)	[0.41–0.91]	[0.41–0.91]	[0.00–0.33]	[0–0.23]	[0.57–0.98]	[0.23–0.77]	[0.17–0.71]	[0.08–0.58]
Unknown	21 (52.50%)	22 (55.00%)	13 (32.50%)	7 (17.50%)	38 (95.00%)	26 (65.00%)	23 (57.50%)	20 (50.00%)
40 (17.32%)	[0.36–0.68]	[0.38–0.70]	[0.18–0.49]	[0.07–0.32]	[0.83–0.99]	[0.48–0.79]	[0.40–0.73]	[0.33–0.66]
Mexico	2 (6.25%)	17 (53.13%)	4 (12.50%)	2 (6.25%)	27 (84.38%)	15 (46.88%)	14 (43.75%)	12 (37.50%)
32 (13.85%)	[0.00–0.20]	[0.34–0.70]	[0.03–0.29]	[0.00–0.20]	[0.67–0.94]	[0.29–0.65]	[0.26–0.62]	[0.21–0.56]
Uruguay	3 (75.00%)	0 (0.00%)	0 (0.00%)	0 (0.00%)	3 (75.00%)	3 (75.00%)	3 (75.00%)	2 (50.00%)
4 (1.73%)	[0.19–0.99]	[0–0.60]	[0–0.60]	[0–0.60]	[0.19–0.99]	[0.19–0.99]	[0.19–0.99]	[0.06–0.93]
Total	99 (42.86%)	136 (58.87%)	53 (22.94%)	29 (12.55%)	207 (89.61%)	135 (58.44%)	129 (55.84%)	105 (45.45%)
231 (100%)	[0.36–0.49]	[0.52–0.65]	[0.17–0.28]	[0.08–0.17]	[0.84–0.93]	[0.51–0.64]	[0.49–0.62]	[0.38–0.52]

### 3.2. Mouse bioassay

No *T. gondii* strain was isolated following inoculation of 118 horse meat samples in mice, including 97 with positive MC-PCR results and 21 with negative MC-PCR results (Supplementary material).

### 3.3. Cut-off determination for MAT and ELISA and level of agreement between tests

**Figure 1.**
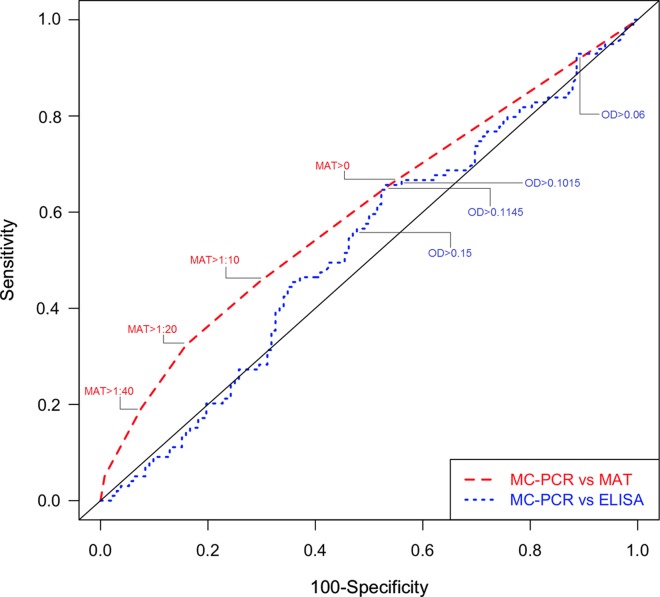
Receiver operating characteristics (ROC) analysis of ELISA and MAT vs. MC-PCR in meat juice samples.

The ROC curves and calculations of sensitivity (Se) and specificity (Sp) allowed us to identify the most suitable cut-offs that represented the best balance between sensitivity and specificity for the two serological tests MAT and ELISA in horse meat samples (Fig. 1). The best balance between sensitivity and specificity was observed at cut-offs MAT > 0 (sensitivity of 66% and specificity of 46%), OD > 0.1015 (sensitivity of 66% and specificity of 47%), and OD > 0.1145 (sensitivity of 62% and specificity of 48%) for ELISA in horse meat samples (Fig. 1 and Table 2). The area under the ROC curve ranged from 0.52 for ELISA to 0.60 for MAT, representing an acceptable level of precision. Other ELISA cut-offs such as OD > 0.06 and OD > 0.15 were subsequently identified by crossing ROC curve results with different MAT cut-offs at > 1:10, > 1:20, and > 1:40, and were also included in our additional calculations. The serial dilutions of the positive horse serum THI1813 showed that the identified cut-off values of ELISA (OD > 0.06, OD > 0.1015, OD > 0.1145, and OD > 0.15) were consistent with the negativity limits of the positive control (data not shown).

**Table 2.** Statistical measures of the performance of ELISA (OD) and MAT with different cut-offs compared with MC-PCR for detecting *T. gondii* in horse meat samples.

	MC-PCR* vs. OD > 0.06	MC-PCR* vs. OD > 0.1015	MC-PCR* vs. OD > 0.1145	MC-PCR* vs. OD > 0.15	MC-PCR* vs. MAT > 0	MC-PCR* vs. MAT > 1:10	MC-PCR* vs. MAT > 1:20	MC-PCR* vs. MAT > 1:40
Sensitivity, %	90.91%	65.66%	61.62%	49.49%	65.66%	45.45%	32.32%	19.19%
(95% CI)	(0.85–0.97)	(0.56–0.75)	(0.52–0.71)	(0.40–0.59)	(0.56–0.75)	(0.36–0.55)	(0.23–0.42)	(0.11–0.27)
Specificity, %	11.36%	46.97%	48.48%	57.58%	46.21%	70.45%	84.09%	92.42%
(95% CI)	(0.06–0.17)	(0.38–0.55)	(0.40–0.57)	(0.49–0.66)	(0.38–0.55)	(0.63–0.78)	(0.78–0.90)	(0.88–0.97)
PPV, %	43.48%	48.15%	47.29%	46.67%	47.79%	53.57%	60.38%	65.52%
(95% CI)	(0.37–0.50)	(0.40–0.57)	(0.39–0.56)	(0.37–0.56)	(0.39–0.56)	(0.43–0.64)	(0.47–0.74)	(0.48–0.83)
NPV, %	62.50%	64.58%	62.75%	60.32%	64.21%	63.27%	62.36%	60.40%
(95% CI)	(0.43–0.82)	(0.55–0.74)	(0.53–0.72)	(0.52–0.69)	(0.55–0.74)	(0.55–0.71)	(0.55–0.69)	(0.54–0.67)
Kappa coefficient	0.02	0.12	0.09	0.07	0.11	0.16	0.17	0.12
McNemar test	*P* < 0.1%	*P* < 0.1%	*P* < 1%	NS	*P* < 0.1%	NS	*P* < 0.1%	*P* < 0.1%

* MC-PCR is the reference test; PPV is the positive predictive value; NPV is the negative predictive value; NS means not significant.

The kappa values used to estimate the agreement between MC-PCR vs. ELISA and MC-PCR vs. MAT for the identified cut-offs were low with a range between 0.02 and 0.12 for the ELISA and between 0.11 and 0.17 for the MAT (Table 2). These results indicate a poor agreement between MC-PCR vs. ELISA and MC-PCR vs. MAT. The McNemar test was significant for the cut-offs OD > 0.06, OD > 0.1015, OD > 0.1145, and MAT > 0 (Table 2). At these cut-off values, the discordances were significantly more frequent in samples with negative results with MC-PCR and positive results with ELISA or MAT than in samples with positive results with MC-PCR and negative results with ELISA or MAT. At cut-off values OD > 0.15 and MAT > 1:10, the McNemar test was not significant (Table 2), which means that the paired discordances (samples negative with MC-PCR and positive with ELISA or MAT vs. samples positive with MC-PCR and negative with ELISA or MAT) occurred randomly. At the MAT > 1:20 and MAT > 1:40 cut-off values, the McNemar test was significant (Table 2), and the discordances were significantly more frequent in samples with positive results with MC-PCR and negative results with MAT than in samples with negative results with MC-PCR and positive results with MAT. This result showed that the MAT > 1:20 and MAT > 1:40 cut-offs have poor sensitivity for detecting horse meat samples infected with *T. gondii*.

### 3.4. Prevalence data

According to MC-PCR results, *T. gondii* DNA was found in 99 (43%) of 231 horse meat samples (Table 1). By using the serological MAT and ELISA tests, the *T. gondii* prevalence varied from 13 to 90% depending on the cut-off used (Table 1). The three cut-offs for MAT and ELISA that were determined by our statistical analysis as having the best balance between sensitivity and specificity showed a similar prevalence with both techniques: 59% (MAT > 0), 58% (OD > 0.1015), and 56% (OD > 0.1145).

## 4. Discussion

Our primary goal was to assess the risk of *T. gondii* infection after consumption of horse meat based on recent reports in France of several cases of severe toxoplasmosis after consumption of horse meat imported from South and North America [34]. Seroprevalence data can give an indication of the risk of human toxoplasmosis by eating meat from one animal species only if the detection of specific antibodies and the presence of tissue cysts have a strong correlation in this animal species [33]. This correlation is excellent in some animal species such as pigs and sheep [17, 23], poor in other animal species such as cattle [33], and totally unknown in the vast majority of animal species. In numerous animal species, especially wild ones, there is no gold standard for estimating *T. gondii* seroprevalence and the performances of the usual serological tests used for detecting *T. gondii* antibodies, such as ELISA, DAT, IFAT, IHAT, LAT, MAT, and SFDT, have never been evaluated. However, it is common practice to assess *T. gondii* seroprevalence by using one test with an arbitrary cut-off and without any data on the sensitivity, specificity, and agreement with other tests. Taking bats as an example, a study in China reported *T. gondii* seroprevalence data in different species of bats by using the MAT with a cut-off at 1:25, without any explanation on why they used this test at this cut-off, probably because they read that it was validated in pigs [13]. However, bats are not pigs and what is true for one animal species is not true for another. A Brazilian study tested the agreement with Cohen’s kappa coefficient between MAT and IFAT in bat sera [7]. The authors showed that the agreement was poor and could not evaluate the specificity and sensitivity of both tests for the diagnosis of toxoplasmosis in bat species. With all these limitations, one can wonder how far we are from the real seroprevalence of *T. gondii* in bats in the Chinese study.

*T. gondii* seroprevalence studies are much more abundant in horses than in bats in the literature [38], but they face the same problem: there is no gold standard – nothing is known about test performances and agreement between tests. This issue was raised by Dubey many years ago when he observed no correlation between the DT and the MAT for detecting *T. gondii* in horses slaughtered for food in North America [18]. The conclusion of this study was that an accurate assessment of prevalence was not possible until additional studies are carried out to determine the sensitivity and specificity of the various serological tests for toxoplasmosis in horses. We evaluated the performances of MAT and ELISA serological tests in meat juice from 231 horse meat samples by comparing them with the detection of *T. gondii* DNA by MC-PCR in these samples. For the evaluation of the serological tests in horse meat samples, MC-PCR was considered as the reference test in this study because the detection of *T. gondii* DNA in horse meat samples always reveals, in the absence of cross contamination, the presence of *T. gondii*. MC-PCR is a newly developed technique with a much higher sensitivity than isolation of total DNA from a fraction of the crude extract [32]. However, the absence of detection of *T. gondii* DNA does not necessarily mean that the horse was not infected by *T. gondii*, because the distribution of tissue cysts may be low and inhomogeneous in muscle samples [33]. Though sensitivity of MC-PCR is a matter of concern as a reference test in this study with the relatively small sample used (150 g), it can be considered acceptable with a detection limit estimated at 230 parasites per 100 g of meat, making it possible to detect one cyst of *T. gondii* in 100 g of meat [32].

The conventional gold standard for detecting *T. gondii* cysts in tissues is bioassay in mice or cats [23]. We used bioassay in mice in our study not as a reference test but in order to isolate *T. gondii* strains from horse meat for further genotyping. Because mouse bioassay is time-consuming, we inoculated the horse meat samples that were positive with the MC-PCR assay as a priority. All the bioassays were negative; it was not possible to isolate a single strain following mouse bioassay of 118 horse meat samples. This demonstrates that bioassays in mice are far less sensitive than MC-PCR for detecting *T. gondii* in horse meat samples and validates our choice of MC-PCR as the reference test. A concern about *T. gondii* viability due to excessive delay between the slaughter of the horses and the bioassay of meat samples may be addressed to explain this absence of strain isolation in mice, even though tissue cysts are relatively resistant to changes in temperature and remain infectious in refrigerated (1 to 4 °C) carcasses or minced meat for up to 3 weeks [12]. Testing muscles from fresh carcasses collected in abattoirs would probably greatly enhance the sensitivity of bioassay. Another explanation can be found in the Ct (threshold cycle) values of MC-PCR that were very high for the horse meat samples (median = 38.59), indicating a very low DNA concentration which is known to be associated with poor sensitivity in mouse bioassays [21]. Direct genotyping in horse meat samples with microsatellite markers was not attempted because of the insufficient amount of *T. gondii* DNA to allow PCR-based amplification of single-copy markers [1, 32]. Like in cattle, it appears to be very difficult to isolate strains from horse meat probably because the distribution of cysts is very low in skeletal muscles [2, 16]. Other horse tissues such as brain, heart, or diaphragm collected in abattoirs may have a higher *T. gondii* cyst load than in skeletal muscles and may enhance the sensitivity of bioassay as shown in one study in Egypt [36].

Based on kappa values, our results indicate poor agreement between MC-PCR and ELISA or MAT at different cut-offs identified by ROC curves analysis in horse meat samples. The prevalence of *T. gondii* in 231 horse meat samples was 43% with MC-PCR in our study. By using the serological tests MAT and ELISA, the *T. gondii* prevalence varied considerably, from 13 to 90%, depending on the cut-off used. Our analysis identified three cut-offs for MAT and ELISA as having the best balance between sensitivity and specificity: OD > 0.1015 and OD > 0.1145 for ELISA and MAT > 0. However, at these cut-offs, the sensitivity reached a maximum of 66% and the specificity a maximum of 48% which is very low in comparison with values for other animal species such as pigs or sheep in which sensitivity and specificity values are above 85% for both MAT and ELISA [23, 29, 35]. With such low performance of MAT and ELISA in horses, we do not recommend use of serological tests or serological data from the literature to assess the risk of toxoplasmosis after consumption of horse meat. Based on the fact that no viable strain was isolated after mouse bioassay, our study suggests that this risk is low but should not be neglected in France because horse meat is imported from Latin American countries where atypical and pathogenic strains are more common than in Europe, and because eating raw or undercooked meat is common in France. The health authorities should undertake a large-scale survey on a representative sample of imported horses slaughtered for food to assess the risk of toxoplasmosis for consumers. Rather than using retail meat packaged for sale from supermarkets, the detection of *T. gondii* by bioassay should be done on fresh carcasses to maximize the viability of the parasite. To avoid any risk of severe toxoplasmosis, the consumer must cook the horse meat by reaching an internal temperature above 70 °C in all parts of the meat, which will kill the cysts of *T. gondii* if present.

## Online material

### PDF version of all Online Materials


