# Supplementary material for: Detection of Toxoplasma gondii DNA in horse meat from supermarkets in France and performance evaluation of two serological tests
Source: Parasite. 2015 Mar 25;22:14. doi: 10.1051/parasite/2015014 (PMC4374124; doi:10.1051/parasite/2015014)
Supplement: Supplementary file 1 [file parasite150019-parasite-22-14-Material.pdf]

PARASITE
Available online at:
www.parasite-journal.org

SUPPLEMENTARY MATERIAL OPEN ACCESS

Detection of *Toxoplasma gondii* DNA in horse meat from supermarkets in France and performance evaluation of two serological tests

Abdelkrim Aroussi[1], Philippe Vignoles[1], François Dalmay[2], Laurence Wimel[3], Marie-Laure Dardé[1,4], Aurélien Mercier[1], and Daniel Ajzenberg[1,4,*]

[1] INSERM, Univ. Limoges, CHU Limoges, UMR-S 1094, Laboratoire de Parasitologie, 87000 Limoges, France
[2] INSERM, Univ. Limoges, CHU Limoges, UMR-S 1094, Institut d'Epidémiologie Neurologique et de Neurologie Tropicale, 87000 Limoges, France
[3] Institut Français du Cheval et de l'Équitation, Station Expérimentale, Domaine de la Valade, 19370 Chamberet, France
[4] Toxoplasma Biological Resource Center, CHU Limoges, 87042 Limoges, France

Parasite 2015, 22, 14, DOI: 10.1051/parasite/2015014

Sample	Geographic origin	OD value ELISA	MAT (dilution)	Mice bioassay (date)	MC-PCR Date, (Ct value)	MAT in mice
1	Argentina	0.1698	Positive (1:10)	Done (1/31/2013)	2/20/2013 (41.17)	Negative
2	Mexico	0.17875	Positive (1:40)	Done (1/30/2013)	2/20/2013 (Negative)	Negative
3	Canada	0.1265	Positive (1:10)	Done (1/31/2013)	2/20/2013 (Negative)	Negative
4	Argentina	0.6608	Positive (1:20)	Done (1/30/2013)	2/20/2013 (36.20)	Negative
5	Argentina	0.1915	Positive (1:10)	Done (1/30/2013)	2/20/2013 (39.85)	Negative
6	Canada	0.0664	Positive (1:10)	Done (2/07/2013)	2/10/2013 (Negative)	Negative
7	Canada	0.0931	Positive (1:10)	Not done	2/10/2013 (Negative)	Not done
8	Canada	0.13495	Positive (1:10)	Done (1/30/2013)	2/20/2013 (40.14)	Negative
9	Canada	0.11245	Positive (1:10)	Not done	2/20/2013 (Negative)	Not done
10	Canada	0.7202	Positive (1:40)	Done (1/31/2013)	2/20/2013 (Negative)	Negative
11	Mexico	0.0622	Positive (1:10)	Not done	2/22/2013 (Negative)	Not done
12	France	0.10085	Positive (1:10)	Done (3/14/2013)	3/17/2013 (Negative)	Negative
13	Canada	0.1472	Positive (1:10)	Done (3/14/2013)	3/17/2013 (Negative)	Negative
14	France	0.0674	Positive (1:10)	Done (3/25/2013)	3/28/2013 (Negative)	Negative
15	Mexico	0.0544	Positive (1:100)	Done (3/15/2013)	3/18/2013 (Negative)	Negative
16	Mexico	0.0771	Positive (1:20)	Done (3/19/2013)	5/07/2013 (Negative)	Negative
17	Canada	0.36375	Positive (1:40)	Done (3/19/2013)	5/07/2013 (Negative)	Negative
18	Argentina	0.18455	Positive (1:10)	Not done	5/07/2013 (Negative)	Not done
19	Argentina	0.2686	Positive (1:20)	Not done	5/07/2013 (Negative)	Not done
20	Canada	0.06355	Positive (1:20)	Done (3/19/2013)	5/07/2013 (Negative)	Negative
21	Argentina	0.0548	Positive (1:10)	Not done	5/07/2013 (Negative)	Not done
22	France	0.11875	Positive (1:20)	Not done	5/07/2013 (40.33)	Not done
23	Mexico	0.25185	Positive (1:10)	Done (3/25/2013)	3/28/2013 (Negative)	Negative
24	Mexico	0.29595	Positive (1:10)	Done (3/25/2013)	3/28/2013 (Negative)	Negative
25	Unknown	0.10115	Positive (1:20)	Not done	5/07/2013 (Negative)	Not done
26	Canada	0.132	Positive (1:400)	Done (3/12/2013)	3/15/2013 (Negative)	Negative
27	Canada	0.0886	Positive (1:20)	Done (3/12/2013)	3/15/2013 (Negative)	Negative
28	Canada	0.21	Positive (1:100)	Done (3/15/2013)	3/18/2013 (Negative)	Negative
29	Canada	0.25785	Positive (1:40)	Done (3/17/2013)	3/20/2013 (Negative)	Negative
30	Unknown	0.3198	Positive (1:10)	Done (3/25/2013)	3/28/2013 (Negative)	Negative
31	Argentina	0.26715	Positive (1:10)	Not done	5/7/2013 (Negative)	Not done
32	Argentina	0.24395	Positive (1:10)	Not done	5/7/2013 (38.40)	Not done
33	Canada	0.2282	Positive (1:10)	Done (3/12/2013)	3/15/2013 (Negative)	Negative
34	Canada	0.18925	Positive (1:40)	Done (3/17/2013)	3/20/2013 (Negative)	Negative
35	Canada	0.28675	Positive (1:100)	Done (3/17/2013)	3/20/2013 (Negative)	Negative
36	France	0.1839	Positive (1:10)	Done (4/30/2013)	4/30/2013 (43.09)	Negative
37	Argentina	0.22415	Negative	Not done	4/30/2013 (Negative)	Not done
38	Mexico	0.15545	Negative	Not done	4/30/2013 (Negative)	Not done
39	Mexico	0.28705	Negative	Not done	4/30/2013 (Negative)	Not done
40	Canada	0.53955	Negative	Not done	5/7/2013 (Negative)	Not done
41	Canada	0.0893	Negative	Not done	5/7/2013 (Negative)	Not done
42	Canada	0.97195	Negative	Not done	5/7/2013 (Negative)	Not done
43	Canada	0.25675	Negative	Done (5/07/2013)	5/7/2013 (39.66)	Negative
44	Canada	0.0729	Positive (1:10)	Not done	5/7/2013 (Negative)	Not done
45	Canada	0.0677	Negative	Not done	5/7/2013 (Negative)	Not done
46	Unknown	0.10195	Negative	Done (6/05/2013)	6/5/2013 (39.22)	Negative
47	Unknown	0.0729	Positive (1:10)	Done (6/05/2013)	6/5/2013 (39.10)	Negative
48	Unknown	0.0821	Positive (1:10)	Done (6/05/2013)	6/5/2013 (42.18)	Negative
49	Unknown	0.1612	Negative	Not done	6/5/2013 (Negative)	Not done
50	Unknown	0.08135	Negative	Not done	6/5/2013 (Negative)	Not done
51	Unknown	0.3044	Negative	Done (6/05/2013)	6/5/2013 (38.15)	Negative
52	Unknown	0.05275	Negative	Not done	6/5/2013 (Negative)	Not done
53	Unknown	0.25915	Negative	Done (6/05/2013)	6/5/2013 (40.03)	Negative

54	Canada	0.0726	Positive (1:10)	Done (7/29/2013)	7/29/2013 (36.47)	Negative
55	Argentina	0.3148	Negative	Done (7/29/2013)	7/29/2013 (40.91)	Negative
56	Argentina	0.0612	Negative	Done (7/29/2013)	7/29/2013 (41.01)	Negative
57	Argentina	0.0872	Negative	Done (7/29/2013)	7/29/2013 (38.27)	Negative
58	Argentina	0.2098	Positive (1:100)	Done (12/12/2013)	12/11/2013 (38.43)	Negative
59	Argentina	0.0782	Positive (1:100)	Done (12/12/2013)	12/11/2013 (36.44)	Negative
60	Argentina	0.1657	Positive (1:100)	Done (12/12/2013)	12/11/2013 (40.18)	Negative
61	Argentina	0.0901	Negative	Done (12/12/2013)	12/11/2013 (37.09)	Negative
62	Argentina	0.1575	Positive (1:400)	Done (12/12/2013)	12/11/2013 (38.20)	Negative
63	Argentina	0.1893	Negative	Done (12/12/2013)	12/11/2013 (38.14)	Negative
64	Argentina	0.1400	Positive (1:100)	Done (12/12/2013)	12/11/2013 (37.91)	Negative
65	Argentina	0.2628	Positive (1:400)	Done (12/12/2013)	12/11/2013 (37.85)	Negative
66	Argentina	0.4352	Positive (1:100)	Done (12/12/2013)	12/11/2013 (37.54)	Negative
67	Argentina	0.1888	Positive (1:100)	Done (12/12/2013)	12/11/2013 (37.69)	Negative
68	Argentina	0.0722	Negative	Not done	12/19/2013 (Negative)	Not done
69	Argentina	0.0514	Positive (1:100)	Not done	12/19/2013 (Negative)	Not done
70	Argentina	0.5190	Positive (1:10)	Not done	12/19/2013 (Negative)	Not done
71	Argentina	0.1772	Positive (1:20)	Done (12/20/2013)	12/19/2013 (38.46)	Negative
72	Canada	0.0766	Positive (1:10)	Done (12/20/2013)	12/19/2013 (39.04)	Negative
73	Canada	0.0542	Positive (1:20)	Not done	12/19/2013 (Negative)	Not done
74	Argentina	0.1578	Positive (1:20)	Not done	12/19/2013 (Negative)	Not done
75	Argentina	0.1910	Positive (1:10)	Done (12/20/2013)	12/19/2013 (38.98)	Negative
76	Canada	0.0613	Positive (1:40)	Done (12/20/2013)	12/19/2013 (38.23)	Negative
77	Canada	0.0499	Positive (1:400)	Done (12/20/2013)	12/19/2013 (40.68)	Negative
78	Mexico	0.0747	Positive (1:20)	Done (1/09/2014)	1/8/2014 (38.91)	Negative
79	Mexico	0.1887	Positive (1:40)	Done (1/09/2014)	1/8/2014 (38.83)	Negative
80	Mexico	0.0776	Positive (1:20)	Not done	1/8/2014 (Negative)	Not done
81	Mexico	0.0593	Positive (1:20)	Not done	1/8/2014 (Negative)	Not done
82	Mexico	0.1445	Negative	Not done	1/19/2014 (Negative)	Not done
83	Mexico	0.0549	Positive (1:10)	Not done	1/19/2014 (Negative)	Not done
84	Mexico	0.0927	Negative	Not done	1/19/2014 (Negative)	Not done
85	Mexico	0.0588	Negative	Not done	1/19/2014 (Negative)	Not done
86	Mexico	0.0854	Negative	Not done	1/19/2014 (Negative)	Not done
87	Mexico	0.0850	Negative	Not done	1/19/2014 (Negative)	Not done
88	Mexico	0.0580	Positive (1:10)	Not done	1/19/2014 (Negative)	Not done
89	Mexico	0.1621	Negative	Not done	1/19/2014 (Negative)	Not done
90	Mexico	0.1048	Negative	Not done	1/19/2014 (Negative)	Not done
91	Mexico	0.1646	Positive (1:20)	Not done	1/19/2014 (Negative)	Not done
92	Mexico	0.0845	Negative	Not done	1/19/2014 (Negative)	Not done
93	Mexico	0.1370	Positive (1:20)	Not done	1/19/2014 (Negative)	Not done
94	Argentina	0.1324	Negative	Done (2/18/2014)	2/17/2014 (37.94)	Negative
95	Argentina	0.0843	Positive (1:100)	Not done	2/17/2014 (Negative)	Not done
96	Argentina	0.1276	Negative	Not done	2/17/2014 (Negative)	Not done
97	Argentina	0.0844	Negative	Not done	2/17/2014 (Negative)	Not done
98	Argentina	0.0961	Positive (1:20)	Not done	2/17/2014 (Negative)	Not done
99	Argentina	0.0582	Positive (1:20)	Not done	2/17/2014 (Negative)	Not done
100	Argentina	0.0801	Positive (1:100)	Done (2/18/2014)	2/17/2014 (39.43)	Negative
101	Canada	0.0965	Negative	Not done	2/19/2014 (Negative)	Not done
102	Canada	0.1015	Negative	Not done	2/19/2014 (Negative)	Not done
103	Canada	0.8272	Negative	Not done	2/19/2014 (Negative)	Not done
104	Argentina	0.1193	Negative	Not done	2/19/2014 (Negative)	Not done
105	Argentina	0.1660	Positive (1:20)	Not done	2/19/2014 (Negative)	Not done
106	Argentina	0.0515	Negative	Done (2/20/2014)	2/19/2014 (41.89)	Negative
107	Argentina	0.0798	Positive (1:10)	Not done	2/19/2014 (Negative)	Not done

108	Argentina	0.1458	Positive (1:10)	Not done	2/19/2014 (Negative)	Not done
109	Argentina	0.0770	Positive (1:20)	Done (2/20/2014)	2/19/2014 (40.96)	Negative
110	Argentina	0.0671	Positive (1:10)	Not done	2/19/2014 (Negative)	Not done
111	Argentina	0.3352	Negative	Not done	2/19/2014 (Negative)	Not done
112	Argentina	0.1000	Negative	Not done	2/19/2014 (Negative)	Not done
113	Argentina	0.0542	Negative	Not done	2/19/2014 (Negative)	Not done
114	Argentina	0.0679	Negative	Not done	2/19/2014 (Negative)	Not done
115	Argentina	0.0503	Negative	Not done	2/19/2014 (Negative)	Not done
116	Argentina	0.0793	Positive (1:400)	Not done	2/19/2014 (Negative)	Not done
117	Argentina	0.0578	Negative	Not done	2/19/2014 (Negative)	Not done
118	Argentina	0.2389	Negative	Not done	2/19/2014 (Negative)	Not done
119	Argentina	0.0624	Positive (1:10)	Done (2/28/2014)	2/27/2014 (39.65)	Negative
120	Argentina	0.0544	Positive (1:10)	Not done	2/27/2014 (Negative)	Not done
121	Argentina	0.1246	Negative	Not done	2/27/2014 (Negative)	Not done
122	Argentina	0.1788	Positive (1:10)	Done (2/28/2014)	2/27/2014 (39.23)	Negative
123	Argentina	0.1202	Positive (1:10)	Not done	2/27/2014 (Negative)	Not done
124	Argentina	0.5663	Negative	Not done	2/27/2014 (Negative)	Not done
125	France	0.1854	Positive (1:20)	Done (3/09/2014)	3/8/2014 (42.32)	Negative
126	France	0.1723	Positive (1:20)	Done (3/09/2014)	3/8/2014 (41.89)	Negative
127	France	0.4169	Positive (1:20)	Done (3/09/2014)	3/8/2014 (38.62)	Negative
128	Canada	0.7002	Positive (1:40)	Done (3/09/2014)	3/8/2014 (41.27)	Negative
129	Canada	0.5911	Negative	Not done	3/8/2014 (Negative)	Not done
130	Canada	0.4476	Negative	Not done	3/12/2014 (Negative)	Not done
131	Canada	0.7918	Negative	Not done	3/12/2014 (Negative)	Not done
132	Canada	0.4693	Positive (1:40)	Done (3/13/2014)	3/12/2014 (40.96)	Negative
133	Canada	0.8738	Negative	Done (3/13/2014)	3/12/2014 (41.33)	Negative
134	Canada	0.4072	Negative	Not done	3/12/2014 (Negative)	Not done
135	Argentina	0.8260	Positive (1:40)	Done (4/15/2014)	4/14/2014 (35.81)	Negative
136	Argentina	0.7723	Positive (1:100)	Done (4/15/2014)	4/14/2014 (37.61)	Negative
137	Argentina	0.5686	Positive (1:40)	Done (4/15/2014)	4/14/2014 (38.59)	Negative
138	Canada	0.6401	Negative	Done (4/15/2014)	4/14/2014 (38.49)	Negative
139	Argentina	0.0514	Negative	Done (4/15/2014)	4/14/2014 (36.89)	Negative
140	Argentina	0.0474	Positive (1:10)	Not done	4/14/2014 (Negative)	Not done
141	Argentina	0.0606	Positive (1:10)	Done (4/16/2014)	4/14/2014 (37.17)	Negative
142	Argentina	0.1293	Positive (1:20)	Done (4/16/2014)	4/14/2014 (36.25)	Negative
143	Argentina	0.1252	Positive (1:10)	Done (4/16/2014)	4/14/2014 (37.53)	Negative
144	Argentina	0.0811	Positive (1:20)	Done (4/16/2014)	4/14/2014 (38.51)	Negative
145	Argentina	0.0811	Positive (1:20)	Done (4/16/2014)	4/14/2014 (36.56)	Negative
146	Argentina	0.0599	Positive (1:40)	Done (4/16/2014)	4/14/2014 (42.60)	Negative
147	Argentina	0.0619	Positive (1:40)	Done (4/16/2014)	4/14/2014 (37.85)	Negative
148	Argentina	0.4729	Positive (1:40)	Done (4/16/2014)	4/14/2014 (38.22)	Negative
149	Argentina	0.1341	Positive (1:10)	Done (4/24/2014)	4/23/2014 (33.78)	Negative
150	Argentina	1.0333	Positive (1:10)	Done (4/24/2014)	4/23/2014 (35.97)	Negative
151	Argentina	0.0563	Positive (1:10)	Done (4/24/2014)	4/23/2014 (35.53)	Negative
152	Argentina	0.0684	Negative	Done (4/24/2014)	4/23/2014 (35.46)	Negative
153	Argentina	0.0795	Positive (1:10)	Done (4/24/2014)	4/23/2014 (35.63)	Negative
154	Argentina	0.0999	Positive (1:20)	Done (4/24/2014)	4/23/2014 (35.49)	Negative
155	Argentina	0.0670	Positive (1:10)	Done (4/24/2014)	4/23/2014 (35.86)	Negative
156	France	0.0615	Positive (1:10)	Done (4/24/2014)	4/23/2014 (34.90)	Negative
157	France	0.1071	Negative	Done (4/24/2014)	4/23/2014 (36.27)	Negative
158	Argentina	0.2457	Negative	Done (4/24/2014)	4/23/2014 (33.71)	Negative
159	Argentina	0.1650	Negative	Done (4/24/2014)	4/23/2014 (35.91)	Negative
160	Argentina	0.1415	Negative	Done (4/24/2014)	4/23/2014 (35.41)	Negative
161	Unknown	0.7063	Negative	Not done	5/2/2014 (Negative)	Not done

162	Unknown	0.7214	Negative	Not done	5/2/2014 (Negative)	Not done
163	Unknown	1.1785	Positive (1:40)	Not done	5/2/2014 (Negative)	Not done
164	Unknown	1.1358	Negative	Not done	5/2/2014 (Negative)	Not done
165	Unknown	0.1683	Negative	Done (5/03/2014)	5/2/2014 (40.49)	Negative
166	Unknown	0.0529	Positive (1:40)	Done (5/03/2014)	5/2/2014 (40.28)	Negative
167	Unknown	0.3587	Positive (1:100)	Done (5/03/2014)	5/2/2014 (40.55)	Negative
168	Unknown	0.1789	Negative	Done (5/03/2014)	5/2/2014 (39.50)	Negative
169	Unknown	0.4527	Positive (1:100)	Not done	5/2/2014 (Negative)	Not done
170	Unknown	0.1209	Positive (1:20)	Done (5/09/2014)	5/8/2014 (40.23)	Negative
171	Unknown	0.4678	Positive (1:40)	Not done	5/8/2014 (Negative)	Not done
172	Unknown	0.9565	Negative	Done (5/09/2014)	5/8/2014 (45.44)	Negative
173	Unknown	0.1087	Negative	Done (5/09/2014)	5/8/2014 (40.09)	Negative
174	Unknown	0.2025	Positive (1:40)	Not done	5/8/2014 (Negative)	Not done
175	Unknown	0.0604	Negative	Done (5/09/2014)	5/8/2014 (42.39)	Negative
176	Unknown	0.0721	Negative	Not done	5/8/2014 (Negative)	Not done
177	Unknown	0.0633	Positive (1:100)	Not done	5/8/2014 (Negative)	Not done
178	Unknown	0.4034	Negative	Done (4/15/2014)	5/14/2014 (41.05)	Negative
179	Unknown	0.2630	Negative	Done (4/15/2014)	5/14/2014 (38.25)	Negative
180	Unknown	0.0650	Negative	Not done	5/14/2014 (Negative)	Not done
181	Unknown	0.1357	Positive (1:100)	Done (4/15/2014)	5/14/2014 (39.06)	Negative
182	Unknown	0.1689	Positive (1:20)	Not done	5/14/2014 (Negative)	Not done
183	Unknown	0.1532	Positive (1:400)	Done (4/15/2014)	5/14/2014 (41.03)	Negative
184	Unknown	0.0785	Positive (1:40)	Done (4/15/2014)	5/14/2014 (42.37)	Negative
185	Unknown	0.1162	Positive (1:100)	Done (4/15/2014)	5/14/2014 (40.13)	Negative
186	Unknown	0.1070	Positive (1:100)	Done (4/15/2014)	5/14/2014 (40.30)	Negative
187	Unknown	0.0879	Positive (1:100)	Not done	5/14/2014 (Negative)	Not done
188	Unknown	0.0738	Positive (1:100)	Not done	5/14/2014 (Negative)	Not done
189	Unknown	0.0821	Positive (1:100)	Not done	5/14/2014 (Negative)	Not done
190	Argentina	1.0892	Negative	Not done	6/4/2014 (Negative)	Not done
191	Argentina	0.3642	Negative	Not done	6/4/2014 (Negative)	Not done
192	Argentina	0.2218	Negative	Not done	6/4/2014 (Negative)	Not done
193	Allemagne	0.2844	Negative	Not done	6/4/2014 (Negative)	Not done
194	Allemagne	0.3106	Negative	Done (6/05/2014)	6/4/2014 (43.81)	Negative
195	Mexico	0.3858	Negative	Not done	6/4/2014 (Negative)	Not done
196	Argentina	0.1807	Positive (1:100)	Done (6/05/2014)	6/4/2014 (44.71)	Negative
197	Mexico	0.6581	Positive (1:100)	Not done	6/4/2014 (Negative)	Not done
198	Mexico	0.4767	Positive (1:100)	Not done	6/4/2014 (Negative)	Not done
199	Argentina	0.0773	Negative	Not done	6/4/2014 (Negative)	Not done
200	Argentina	0.9201	Positive (1:400)	Not done	6/4/2014 (Negative)	Not done
201	Argentina	0.8578	Positive (1:40)	Not done	6/4/2014 (Negative)	Not done
202	Mexico	0.0997	Positive (1:100)	Not done	6/4/2014 (Negative)	Not done
203	Canada	0.1635	Positive (1:100)	Not done	6/18/2014 (Negative)	Not done
204	Canada	0.1532	Positive (1:100)	Not done	6/18/2014 (Negative)	Not done
205	Canada	0.1925	Positive (1:100)	Not done	6/18/2014 (Negative)	Not done
206	Canada	0.1655	Positive (1:40)	Not done	6/18/2014 (Negative)	Not done
207	Argentina	0.1652	Negative	Not done	6/18/2014 (Negative)	Not done
208	Argentina	0.0975	Negative	Not done	6/18/2014 (Negative)	Not done
209	Argentina	0.1508	Negative	Done (6/19/2014)	6/18/2014 (38.38)	Negative
210	Canada	0.0725	Positive (1:40)	Not done	6/18/2014 (Negative)	Not done
211	Canada	0.0738	Negative	Not done	6/18/2014 (Negative)	Not done
212	Uruguay	0.3389	Negative	Done (6/19/2014)	6/18/2014 (38.57)	Negative
213	Uruguay	0.3724	Negative	Done (6/19/2014)	6/18/2014 (36.67)	Negative
214	Unknown	0.2737	Positive (1:40)	Done (6/19/2014)	6/18/2014 (35.88)	Negative
215	France	0.0802	Positive (1:40)	Done (6/19/2014)	6/18/2014 (39.86)	Negative

216	France	0.1337	Positive (1:100)	Not done	6/18/2014 (Negative)	Not done
217	France	0.0748	Negative	Not done	6/18/2014 (Negative)	Not done
218	Argentina	0.0998	Negative	Not done	6/18/2014 (Negative)	Not done
219	Argentina	0.0767	Negative	Not done	6/18/2014 (Negative)	Not done
220	Argentina	0.1229	Negative	Done (6/19/2014)	6/18/2014 (38.70)	Negative
221	Mexico	0.0783	Negative	Not done	6/18/2014 (Negative)	Not done
222	Mexico	0.0952	Negative	Not done	6/18/2014 (Negative)	Not done
223	Mexico	0.0633	Negative	Not done	6/18/2014 (Negative)	Not done
224	Uruguay	0.0599	Negative	Done (6/19/2014)	6/18/2014 (37.94)	Negative
225	Uruguay	0.1447	Negative	Not done	6/18/2014 (Negative)	Not done
226	France	0.0542	Negative	Done (6/19/2014)	6/18/2014 (37.43)	Negative
227	France	0.0547	Negative	Done (6/19/2014)	6/18/2014 (41.51)	Negative
228	Canada	0.1877	Positive (1:400)	Done (6/19/2014)	6/18/2014 (38.04)	Negative
229	Canada	0.1670	Positive (1:100)	Done (6/19/2014)	6/18/2014 (34.31)	Negative
230	Mexico	0.1759	Negative	Not done	6/18/2014 (Negative)	Not done
231	Argentina	0.0613	Negative	Not done	6/18/2014 (Negative)	Not done
